# Ischaemia with no obstructive coronary arteries

**DOI:** 10.1007/s12471-020-01451-9

**Published:** 2020-08-11

**Authors:** R. E. Konst, J. G. Meeder, M. E. Wittekoek, A. H. E. M. Maas, Y. Appelman, J. J. Piek, T. P. van de Hoef, P. Damman, S. E. Elias-Smale

**Affiliations:** 1 Department of Cardiology, Radboud University Medical Center, Nijmegen, The Netherlands; 2 Department of Cardiology, VieCuri Medical Center, Venlo, The Netherlands; 3 HeartLife klinieken, Utrecht, The Netherlands; 4 Department of Cardiology, Amsterdam UMC, Location VUMC, University of Amsterdam, Amsterdam, The Netherlands; 5 Department of Clinical and Experimental Cardiology, Heart Center, Amsterdam Cardiovascular Sciences, Amsterdam UMC, University of Amsterdam, Amsterdam, The Netherlands

**Keywords:** INOCA, Coronary vascular dysfunction, Microvascular angina, Coronary vasospasm, Gender

## Abstract

Ischaemia with no obstructive coronary arteries (INOCA) is a common ischaemic heart disease with a female preponderance, mostly due to underlying coronary vascular dysfunction comprising coronary microvascular dysfunction and/or epicardial coronary vasospasm. Since standard ischaemia detection tests and coronary angiograms are not suitable to diagnose coronary vascular dysfunction, INOCA is often overlooked in current cardiology practice. Future research, including large outcome trials, is much awaited. Yet, adequate diagnosis is possible and treatment options are available and vital to reduce symptoms and most probably improve cardiovascular prognosis. This review intends to give a brief overview of the clinical presentation, underlying pathophysiology, and the diagnostic and treatment options in patients with suspected INOCA.

## Dutch contribution to the field

- The NVVC guideline on Angina Pectoris without Obstructive Coronary Artery Disease is about to be launched in the Netherlands to guide cardiologists in recognition, diagnosis and treatment of INOCA patients.
- Radboudumc Nijmegen has an unique cardiology outpatient clinic dedicated to patients with suspected INOCA.
- In the Netherlands, multiple medical centres are able to perform invasive coronary reactivity tests to diagnose vasomotor dysfunction as underlying cause of INOCA.
- Various Dutch cardiologists are members of the Coronary Vasomotor Disorders International Study Group (COVADIS).

## Introduction

**Fig. 1:**
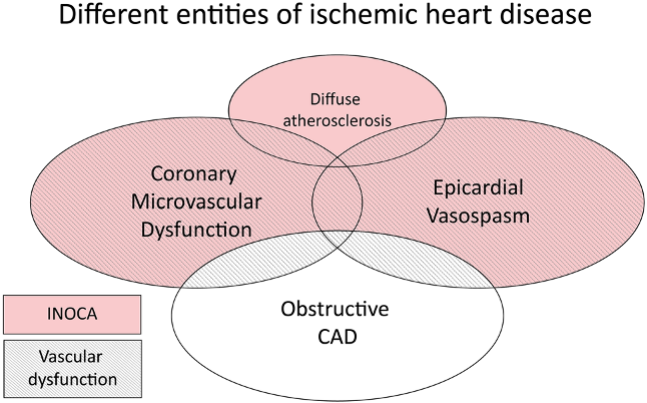
Various entities of ischaemic heart disease. Recommended treatment options for patients with INOCA, based on the results of the CorMicA trial [50]

The majority of patients with anginal symptoms have no obstructive coronary artery disease (CAD) [1]. This group has a female preponderance. In a large Swedish registry, almost 80% of women under 60 years of age with stable angina symptoms had no visible coronary obstructions at angiography compared with 40% of men [2]. Coronary vascular dysfunction appears to be the underlying cause of ischaemia in as much as 59–89% of these so called ‘Ischaemia with No Obstructive Coronary Arteries (INOCA)’ patients [3, 4], and encompasses coronary microvascular dysfunction as well as epicardial coronary vasospasm ([5, 6]; Fig. 1).

Standard ischaemia detection tests and coronary angiograms are not suitable for diagnosing coronary vascular dysfunction [4, 7], but it can be evaluated with an invasive coronary reactivity test (CRT) assessing macrovascular and microvascular coronary artery spasms, coronary flow reserve (CFR) and microvascular resistance [5, 6]. However, as this test is done in relatively few cardiovascular centres worldwide, this ischaemic heart disease is often overlooked and hence undertreated, especially in women.

Treatment options comprise risk factor management, lifestyle measures and antianginal medication [8]. INOCA patients appear to have an adverse cardiovascular prognosis, poor physical functioning and a reduced quality of life [9, 10]. Prognosis is worse in women and in patients with ongoing angina, in those with inducible ischaemia, visible non-obstructive CAD and/or a CFR ≤2.0 [9, 11–14].

This review intends to give a brief overview of the clinical presentation, underlying pathophysiology, and the diagnostic and treatment options of coronary vascular dysfunction, the ischaemic heart disease that is most common cause of INOCA.

## Underlying coronary pathophysiology

In the coronary microvasculature, pre-arterioles and arterioles predominantly govern the resistance to myocardial perfusion, while epicardial arteries (diameter >500 µm) act as capacitance vessels and offer little resistance to flow. The arteriolar tone of coronary microvasculature makes it possible to maintain constant coronary blood flow over a wide range of coronary perfusion pressures by autoregulation. Epicardial arteries, the coronary microvasculature or both can be involved in the pathophysiological processes of ischaemia beyond that of obstructive epicardial coronary artery disease and may contribute, solely or in combination, to a supply-demand mismatch in symptomatic patients.

The coronary endothelium responds to a variety of mechanical and biochemical signalling molecules and either partially or entirely regulates the vasomotor function by releasing a number of endothelium-dependent relaxation factors (e.g. nitric oxide, prostaglandins and endothelium hyperpolarisation factor) [15, 16]. Some of these substances (acetylcholine, histamine, norepinephrine, serotonin) have a simultaneous direct vasoconstrictor effect on the smooth muscle cells that is attenuated or even reversed by the stimulatory effect of a healthy endothelium. An abnormal vasomotion may occur when the coronary endothelium function is impaired, as is frequently encountered in the early stage of atherosclerosis [16, 17].

Coronary vascular dysfunction may be caused by structural and functional abnormalities. Structural abnormalities include microvascular remodelling in arterioles (intimal thickening, proliferation of smooth muscle cell and perivascular fibrosis) and capillary rarefaction, resulting in increased microvascular resistance. Functional abnormalities include endothelium dysfunction resulting in abnormal vasomotor tone as abnormal response to cholinergic vasodilators (e.g. acetylcholine), resulting in impaired coronary blood flow augmentation.

The diagnostic workup of symptomatic patients without obstructive coronary artery diseases (INOCA) includes the evaluation of the aforementioned vascular domains. Administration of vasodilators (most notably acetylcholine) test the endothelium-dependent vasodilator function of the epicardial arteries and coronary microvasculature, while the administration of vasodilators such as adenosine evaluates the endothelium-independent function of the coronary microcirculation expressed as the coronary flow reserve and microvascular resistance [18].

## Symptoms and risk factors

The main symptom caused by coronary vascular dysfunction is angina. Dyspnoea, fatigue or palpitations may occur in addition to, or instead of, angina (angina equivalents). Angina due to coronary vascular dysfunction, especially microvascular coronary dysfunction, may be very difficult to distinguish from ‘classical’ angina due to obstructive CAD as both are mainly exercise-related. However, coronary microvascular dysfunction is more likely if chest pain persists for several minutes to hours after exercise. Furthermore, angina more often occurs after exercise and is more often triggered by mental stress and palpitations compared with obstructive CAD [19]. Angina at rest is often encountered in addition to exercise-provoked chest pain [20]. These attacks of angina at rest imply that an element of vasospasm is present in some patients with coronary microvascular disease [21]. Pure vasospastic/variant angina due to epicardial spasm, in contrast to classical and microvascular angina, is characterised by angina at rest—especially between night and early morning. However, marked diurnal variation in exercise tolerance, with a reduced tolerance in the morning, is also a feature of vasospastic angina. In addition, patients with vascular dysfunction often experience loss of energy and extreme tiredness that may fluctuate over time (days/weeks). This often affects their ability to work, which is a serious problem in this relatively young population. Furthermore, among patients who are very symptomatic, spasm may be triggered by the elevated catecholamines associated with exercise [22, 23].

## Risk factors

Traditional cardiovascular risk factors, especially dyslipidaemia and obesity, play a role in coronary microvascular dysfunction. But these risk factors only partly (<20%) explain the occurrence of coronary microvascular dysfunction [24]. Systemic inflammatory disease, e.g. chronic inflammatory rheumatoid disease, also appears to play an important role [25, 26]. The role of reproductive risk factors as well as premenopausal migraine and Raynaud’s phenomenon is equivocal to date, and more research on this subject is warranted [27].

Except for smoking, many of the conventional risk factors for atherosclerosis are not applicable to vasospastic angina [5]. It may be associated with other vasospastic disorders, such as Raynaud’s phenomenon and migraine headache or its treatment [28, 29]. Besides smoking, hyperventilation, mental stress and drug abuse, such as cocaine use, can trigger attacks of coronary vasospasm through activation of the sympathetic nervous system [30, 31].

## Non-invasive diagnosis of coronary vascular dysfunction

Standard non-invasive stress tests have limited diagnostic accuracy for detecting coronary vascular dysfunction, with an overall sensitivity of only 41% and a specificity of 57% [32]. The extent of ischaemia in non-invasive stress tests has prognostic value in patients with INOCA, but it is important to realise that a normal stress test does not rule out vasomotor dysfunction as a cause of the angina [33]. To identify vascular dysfunction, a comprehensive assessment of the coronary function should be performed, including assessment of coronary microvascular dysfunction and coronary vasospasm. Non-invasive methods that can be applied to assess coronary flow reserve (equivalents) include positron emission tomography imaging [34], cardiac magnetic resonance [35, 36], and transthoracic Doppler echocardiography [37]. An important overall limitation of all the above-mentioned non-invasive measurements is that they can only assess CFR, while data from invasive vasomotor tests show that the majority of patients suspected of INOCA have an abnormal acetylcholine test [4], occurring more frequently than a pathological CFR result [7].

Microvascular spasm can be evaluated using a cold pressor test, but this has been shown to correlate only moderately with invasive spasm testing using acetylcholine [38].

## Invasive coronary reactivity testing

Epicardial coronary vasospasm and coronary microvascular dysfunction can be diagnosed invasively using CRT (Tab. 1; [5, 6]). After excluding fixed obstructive CAD, both macrovascular and microvascular vascular function can be assessed. For this purpose, a comprehensive CRT includes coronary vasospasm provocation testing with intracoronary acetylcholine for evaluation of endothelium-dependent vasomotor function and microvascular or epicardial vasospasm, as well as evaluation of non-endothelium dependent vasomotor function using adenosine for measurement of coronary flow reserve and microvascular resistance. For the diagnosis of vasospasm, the provocative stimulus (typically acetylcholine but ergonovine can be used as an alternative) is administered in a stepwise manner with increasing doses, during which the patient’s symptoms and ECG are closely monitored. At each step and with the occurrence of recognisable chest pain, coronary angiography is repeated to evaluate the presence of angiographic coronary artery spasm. A spasm provocation test is considered positive in the presence of: (i) reproduction of recognisable chest pain, (ii) ischaemic ECG changes, and (iii) >90% vasoconstriction on angiography. If recognisable chest pain occurs during provocation testing with typical ischaemic ECG shifts, but no epicardial spasm, microvascular spasm can be suspected. Furthermore, coronary microvascular dysfunction may be revealed by: (i) an impaired CFR (cut-off values depending on methodology use between ≤2.0 and ≤2.5) (ii) abnormal microvascular resistance or (iii) coronary slow flow phenomenon. CFR and microvascular resistance can be evaluated with a thermodilution-based or Doppler-based intracoronary wire. While coronary thermodilution is considered more feasible in clinical practice, it has a higher intra-observer variability and poorer correlation with absolute myocardial perfusion and structural microvascular abnormalities, but Doppler flow velocity measurements are currently considered technically more challenging resulting more often in lower quality traces [39]. Current research focuses on the improvement and implementation of these methods. With regards to safety and efficacy of CRT, earlier studies have shown that CRT can be performed safely [40]. Since CRT is currently available in a limited number of medical centres, it not achievable to regularly perform it in patients suspected of INOCA. However, the recent CorMicA trial has shown that the routine use of CRT to inform stratified medical therapy is feasible and reduces angina burden in patients with chest pain and no obstructive CAD [41].

**Table 1:** Overview of the criteria for vasospastic and microvascular angina

	Criteria for vasospastic angina	Criteria for coronary microvascular angina
*Symptoms*	Nitrate-responsive angina—during spontaneous episode, with at least one of the following: a. Rest angina—especially between night and early morning b. Marked diurnal variation in exercise tolerance—reduced in morning c. Hyperventilation can precipitate an episode d. Calcium channel blockers (but not beta-blockers) suppress episodes	Symptoms of myocardial ischaemia a. Effort and/or rest angina b. Angina equivalents (i.e. shortness of breath)
*Absence of obstructive CAD*	Not necessary	Absence of obstructive CAD (>50% diameter reduction or FFR <0.80) by a. Coronary CTA b. Invasive coronary angiography
*Myocardial ischaemia*	Transient ischaemic ECG changes—during spontaneous episode, including any of the following in at least two contiguous leads: a. ST-segment elevation ≥0.1 mV b. ST-segment depression ≥0.1 mV c. New negative U waves	Objective evidence of myocardial ischaemia a. Ischaemic ECG changes during an episode of chest pain b. Stress-induced chest pain and/or ischaemic ECG changes in the presence or absence of transient/reversible abnormal myocardial perfusion and/or wall motion abnormality
*Impaired vascular function*	Coronary artery spasm—defined as transient total or subtotal coronary artery occlusion (>90% constriction) with angina and ischaemic ECG changes either spontaneously or in response to a provocative stimulus (typically acetylcholine, ergonovine, or hyperventilation)	Evidence of impaired coronary microvascular function a. Impaired coronary flow reserve (cut-off values between ≤2.0 and ≤2.5 depending on used methodology) b. Coronary microvascular spasm, defined as reproduction of symptoms, ischaemic ECG shifts but no epicardial spasm during acetylcholine testing c. Abnormal coronary microvascular resistance indices (e.g. IMR >25) d. Coronary slow flow phenomenon, defined as TIMI frame count <25
*Definite diagnosis*	Nitrate-responsive angina is evident during spontaneous episodes and either the transient ischaemic ECG changes during the spontaneous episodes or coronary artery spasm criteria are fulfilled	All criteria are present for a diagnosis of microvascular angina
*Suspected diagnosis*	Nitrate-responsive angina is evident during spontaneous episodes but transient ischaemic ECG changes are equivocal or unavailable and coronary artery spasm criteria are equivocal	Symptoms and absence of obstructive CAD with either evidence of myocardial ischaemia or impaired vascular function

Based on the COVADIS criteria for vasospastic disease [5] microvascular angina [6]

*CAD* coronary artery disease, *FFR* fractional flow reserve, *CTA* computed tomography angiography, *IMR* index of microvascular resistance, *TIMI* Thrombolysis in Myocardial Infarction

In the above-mentioned CorMicA trial, coronary vasomotor dysfunction was diagnosed in 89% of the patients [42]. A study in 1379 consecutive INOCA patients (mean age 62 years, 42% male) with a comparable acetylcholine test to the CorMicA trial reported 813 patients (59%) with a pathological acetylcholine test, 33% for microvascular spasm and 26% for epicardial vasospasm. In 12% of patients the test was negative, in the other 29%, the test result was equivocal. A pathological test was more common in females (70% vs. 43%; *p* < 0.001). This study did not, however, look into coronary flow reserve and microvascular resistance [4].

## Therapeutic options

### Risk factor management and lifestyle modifications

Strict risk factor management plays a pivotal role in the treatment of INOCA [8]. Adequate lifestyle measures, such as smoking cessation, healthy eating and physical activity, promote well-being in patients with INOCA, especially in women [43]. Special exercise programs and cardiac rehabilitation may help achieve long-term lifestyle change. Stress is an important luxating factor for anginal symptoms and needs special attention. Behavioural therapy and meditation can be helpful in stress management and life-balance.

### Disease-modifying therapy

Statins and ACE inhibitors are recommended, as disease-modifying agents, due to their beneficial effect on coronary endothelial function [44].

### Anti-anginal therapy (Fig. 2)

#### Microvascular angina

Beta-blockers should be considered as first-line treatment in patients with an elevated heart rate during rest or low-workload exercise and exercise-related symptoms [45]. However, they should be avoided in patients with proven or suspected coronary spasm, such as in those with symptoms at rest [46]. In these patients, calcium channel blockers such as diltiazem are preferred as first-choice treatment [44], as this drug is effective in both patients with coronary microvascular dysfunction and coronary spasm [47]. If treatment with beta-blockers is not effective enough, calcium channel blockers can be added [45].

**Fig. 2:**
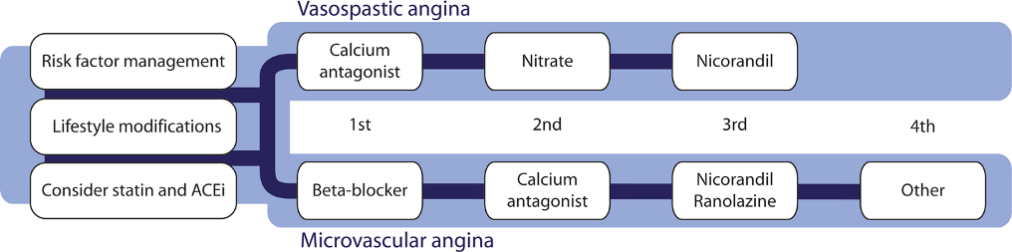
Recommended treatment strategies for patients with INOCA, based on the results of the CorMicA trial [50]. Other options for the treatment of microvascular angina include imipramine and transcutaneous electrical nerve stimulation (TENS). (INOCA ischaemia and no obstructive coronary arteries, CAD coronary artery disease.)

In patients with refractory symptoms, intolerance or contraindications for beta-blockers and/or calcium channel blockers, treatment options include nicorandil and ranolazine [45]. These can be used along with the first-line treatment [45]. Nicorandil is often more effective than long-acting nitrates, since it affects both the vascular smooth muscle cells and nitric oxide production and has a more pronounced effect on the microvasculature [46, 48]. While long-acting nitrates can be added as third-line treatment [45] worsening of endothelial dysfunction is a potential complication and research suggests it is only effective in about half of the patients [45, 46]. Ranolazine, a late sodium channel inhibitor, has been proven effective in patients with CFR <2.5 [49].

In case of persistent symptoms despite the aforementioned therapies, alternative treatment including low-dose imipramine, a tricyclic antidepressant, to treat abnormal cardiac nociception might be considered as third-line therapy [50]. For in-depth options for other third-line options, we refer to a recent comprehensive overview on this topic [51]. Furthermore, non-pharmacological treatment with transcutaneous electrical nerve stimulation (TENS) may be considered as an option to offer for pain control by neurostimulation [45].

#### Vasospastic angina

First-line antianginal treatment mainly consists of calcium channel blockers. In patients with more severe or refractory symptoms, long-acting nitrates can be added [45]. Alternatively, the combination of two calcium channel blockers (dihydropyridine and non-dihydropyridine) can be considered [45]. Nicorandil may also suppress attacks and can therefore be considered as additional treatment to the standard treatment regime for symptom control [50]. Beta-blockers, especially non-selective ones like propranolol, should be avoided since they can exacerbate vasospasm [52].

## Conclusion

INOCA is a common, but often overlooked ischaemic heart disease, mostly due to underlying coronary vascular dysfunction. Adequate diagnosis and treatment is challenging but vital to ameliorate symptoms and prognosis. Further research, taking into account the various underlying pathophysiological mechanisms, is warranted to improve patient tailored diagnostic and therapeutic options.
